# Disentangling racing thoughts from mind wandering in adult attention deficit hyperactivity disorder

**DOI:** 10.3389/fpsyg.2023.1166602

**Published:** 2023-09-04

**Authors:** Emilie Martz, Luisa Weiner, Anne Bonnefond, Sébastien Weibel

**Affiliations:** ^1^INSERM U1114, Université de Strasbourg, Strasbourg, France; ^2^Laboratoire de Psychologie des Cognitions, University of Strasbourg, Strasbourg, France; ^3^Department of Psychiatry, University Hospital of Strasbourg, Strasbourg, France

**Keywords:** adult ADHD, racing thoughts, spontaneous mind wandering, deliberate mind wandering, factor analysis

## Abstract

**Introduction:**

Mental restlessness reported by adult with Attention Deficit Hyperactivity Disorder (ADHD) has been mainly explained by excessive mind wandering. However, the description of a mind constantly on the go is also akin to racing thoughts, predominantly described in bipolar disorder. This paper aimed at disentangling mind wandering from racing thoughts in adult with ADHD. Associations between those mental phenomena and the ADHD symptomatology were also investigated.

**Methods:**

To this aim, 84 adults with ADHD completed self-reported questionnaires, including the Mind Wandering-Deliberate and Mind Wandering-Spontaneous questionnaires, the Racing and Crowded Thoughts Questionnaire and the Daydreaming Frequency Scale. Factorial analysis and multiple linear regressions were performed.

**Results:**

The factor analysis yielded a two-factor solution. The first factor encompassed the three facets of racing thoughts and was predicted by emotional lability. The second comprised deliberated-MW, spontaneous-MW and daydreaming, but was neither related to the ADHD symptoms, nor functional impairment.

**Discussion:**

These findings suggest that MW and racing thoughts are two distinguishable mental phenomena. Racing thoughts appear to be a relevant hypothesis to explain the mental restlessness in adult ADHD.

## 1. Introduction

Attention Deficit Hyperactivity Disorder (ADHD) is the most common neurodevelopmental disorder affecting up to 4% of the adult population (Fayyad et al., 2007; Simon et al., 2009). ADHD diagnosis is based on behavioral symptoms, comprising persistent inattention, motor hyperactivity and impulsivity (Kooij Bejerot et al., 2010; American Psychiatric Association, 2013). However, difficulties experienced by adults with ADHD exceed these observable symptoms. For instance, adults with ADHD deal with increased attentional difficulties and more internalized restlessness in comparison with children with ADHD (Faraone et al., 2000; Adler and Cohen, 2004; Zalsman and Shilton, 2016), but also compared to healthy controls (Weyandt et al., 2016; Guo et al., 2021). Among the invisible symptoms, an overwhelming mental restlessness is frequently reported by patients (Downey et al., 1997; Asherson, 2005; Weyandt et al., 2016). Adults with ADHD describe their thoughts as “constantly on the go,” with multiple thoughts occurring at the same time and ideas flitting from one topic to another (Asherson, 2005; Asherson et al., 2016; Mowlem et al., 2016; Weyandt et al., 2016; Sedgwick et al., 2019).

To date, the subjective experience of mental restlessness in adults with ADHD is mainly hypothesized to be underpinned by excessive mind wandering (MW) (Asherson et al., 2016) although few studies have been conducted in patient samples. MW is defined as moments when attention shifts from the perceptual world (i.e., the ongoing task) to focus on the internal world (one’s thoughts and feelings) (Shaw and Giambra, 1993; Franklin et al., 2014; Seli et al., 2015; Smallwood and Schooler, 2015). Importantly, these shifts of attention can occur intentionally, i.e., deliberately, or unintentionally, i.e., spontaneously (Carriere et al., 2013). In adults with ADHD, studies measuring trait-MW with self-reported questionnaires and those assessing state-MW using experience sampling methods have found that MW, especially spontaneous MW episodes, occur more frequently in comparison with healthy subjects (Shaw and Giambra, 1993; Biederman et al., 2019; Bozhilova Cooper et al., 2020; Madiouni et al., 2020; Alperin et al., 2021). These findings seem coherent considering the failure of executive control theory of MW and the overall cognitive deficits observed in ADHD, in particular the attentional and executive deficits which are the core of the disorder (Boonstra et al., 2005; Seidman, 2006; Pazvantoğlu et al., 2012; Cheung et al., 2016; Mohamed et al., 2021). Indeed, according to the failure of executive control theory, spontaneous MW episodes occur when the executive control resources are depleted and no longer enable the individual to maintain attention on the ongoing task. Consequently, excessive spontaneous MW is more likely to be involved in the mental restlessness reported by adults with ADHD than deliberate MW. However, other spontaneous thinking peculiarities that may account for mental restlessness have been reported in adult ADHD – i.e., racing thoughts –, but have been less studied.

Racing thoughts are defined as the subjective feeling of rapid and numerous thoughts (Piguet et al., 2010; Weiner et al., 2018). Racing thoughts have been recently put forward as an alternative account to explain the ceaseless mental activity of adults with ADHD (Martz et al., 2021, 2022). Interestingly, akin to MW, racing thoughts have been identified as a multi-faced concept, including three characteristics: (i) thought overactivation, referring to the feeling of a rapid train of thoughts, (ii) burden of thought overactivation, implying the unpleasant feeling of numerous overlapping thoughts, and (iii) thought overexcitability, which is related to distractibility (Piguet et al., 2010; Weiner et al., 2018). Although racing thoughts are typically seen as pathognomonic of (hypo) manic thinking in bipolar disorder (Goodwin and Jamison, 2007), adults with ADHD have been found to report increased rates of racing thoughts in comparison to hypomanic patients with bipolar disorder (Martz et al., 2021).

Differences can be found between MW and racing thoughts, especially regarding their associations with the ADHD symptomatology. Contrary to deliberate MW, spontaneous MW have been found to be mainly associated with inattention, but also with motor restlessness and impulsivity, in both healthy subjects and adults with ADHD (Smallwood and Schooler, 2006; Cheyne et al., 2011; Franklin et al., 2014; Seli et al., 2015; Biederman et al., 2017; Arabacı and Parris, 2018). In ADHD, spontaneous MW has also been related to emotional lability (Biederman et al., 2019), which is considered to be an additional core symptom of the disorder (Shaw Stringaris et al., 2014; Hirsch et al., 2018; Weibel et al., 2022). Similarly, racing thoughts were found to be specifically associated with motor hyperactivity, hyperarousal and emotional lability in adults with ADHD (Martz et al., 2021). However, inattention did not predicted any of the three racing thoughts dimensions. Furthermore, from a neuropsychological standpoint, racing thoughts have been associated with semantic overactivation (Martz et al., 2022), whereas attentional control is hypothesized to underlie increased MW (McVay and Kane, 2010; Smallwood and Schooler, 2015). Regarding the impact of MW and racing thoughts on patients’ functioning, results are less straightforward. Some studies highlight the contribution of state but also trait MW to self-reported functional difficulties and to altered performances on cognitive tasks (Biederman et al., 2019; Bozhilova Cooper et al., 2020), but not all (Madiouni et al., 2020; Alperin et al., 2021). Moreover, no study specifically investigated the impact of racing thoughts on patients’ difficulties, although a significant correlation was found between racing thoughts and insomnia severity in a study by Martz et al. (2021).

Overall, similarities and dissimilarities can be pointed out between MW and racing thoughts. However, to date, no study has simultaneously explored MW and racing thoughts, raising the question of the overlap between the concepts. On the one hand, considering the similarities, it can be assumed that racing thoughts are equivalent to MW. On the other hand, considering the differences, racing thoughts and MW may be considered as co-occurring mental phenomena contributing to mental restlessness in adult ADHD. To focus on this issue, this study aimed to investigate, simultaneously, self-reported MW and racing thoughts in adult ADHD. To do so, adults with ADHD completed self-report questionnaires measuring MW and racing thoughts in order to specify whether these two concepts are dissociable in ADHD. Moreover, we were interested in the links between thinking peculiarities and ADHD symptomatology and functional impairment. In particular, we expected MW to be predicted by inattention symptoms, whereas hyperactivity and emotional lability should contribute to racing thoughts.

## 2. Methods

### 2.1. Participants

The sample included 84 adults with ADHD aged 18–57 (*M* = 32.48 years; *SD* = 10.23; 57.12% female). Participants were recruited from the outpatient psychiatry clinics of the University Hospital of Strasbourg. ADHD and comorbidities diagnoses were established by senior psychiatrists based on the DSM-5 criteria (American Psychiatric Association, 2013). No exclusion criteria were applied. Demographic data, comorbidities and treatment were recorded at the diagnostic assessment. Detailed demographic data are provided in Table 1. Subjects provided written informed consent prior to inclusion in the study in accordance with the Declaration of Helsinki. This study was approved by the regional ethics committee of the South of France (CPP SUD MEDITERRANEE II; February 19, 2018; reference number: 2017-A01618-45).

**Table 1:** Demographic characteristics of adults with ADHD.

Variables	Total sample (*n* = 84)
Age, Mean (SD)	32.48 (10.23)
Gender, *N* (%)	34 M (40.48%)
	48 F (57.12%)
	2 transgender (2.38%)
Years of education, Mean*(SD)*	14.07 (2.48)
Comorbidities, *N* (%)	
Past major depression episode	27 (32.14%)
Current major depression episode	3 (3.57%)
Bipolar disorder	13 (15.48%)
Cyclothymia	7 (8.33%)
Anxiety disorder	15 (17.86%)
Obsessive compulsive disorder	2 (2.38%)
Borderline personality disorder	6 (7.14%)
Other personality disorder	2 (2.38%)
Autism spectrum disorder	6 (7.14%)
Treatments, *N* (%)	
Antidepressants	14 (16.67%)
Anxiolytics	6 (7.14%)
Hypnotics	0 (0.00%)
Mood stabilizers	7 (8.33%)
Antipsychotics	3 (3.57%)
Psychostimulants	10 (11.90%)
Other treatments	7 (8.33%)
Questionnaires scores, Mean (SD)	
SR-WRAADDS – Attention/Disorganization	24.37 (5.08)
SR-WRAADDS – Hyperactivity/Restlessness	7.20 (3.27)
SR-WRAADDS – Impulsivity/Emotional outbursts	10.91 (4.91)
SR-WRAADDS – Emotional Lability	19.01 (4.56)
SR-WRAADDS – Opposition defiant behaviors	18.46 (7.56)
SR-WRAADDS – Additional symptoms	16.27 (4.54)
SR-WRAADDS – Academic difficulties	9.08 (4.29)
SR-WRAADDS – Social skills	24.42 (8.73)
RCTQ-13 – Total	34 (11.21)
RCTQ-13 – Thought overactivation	10.99 (3.91)
RCTQ-13 – Burden of thought overactivation	10.05 (4.21)
RCTQ-13 – Thought overexcitability	12.96 (4.49)
MW-deliberate	21.35 (4.80)
MW-spontaneous	23.59 (3.54)
Daydreaming Frequency Scale	51.85 (5.21)

SR-WRAADDS, Self-Reported Wender-Reimherr Adult Attention Deficit Disorder Scale; RCTQ-13, Racing and Crowded Thoughts Questionnaire-13 items; MW, mind wandering.

### 2.2. Questionnaires

The Racing and Crowded Thoughts Questionnaire-13-item (RCTQ-13) (Weiner et al., 2019) is a 13-item self-reported questionnaire assessing 3 facets of racing thoughts during the last 24 h – i.e., thought overactivation, burden of thought overactivation and thought overexcitability. Items are scored on a 5-point Likert scale ranging from 0 (“not at all”) tot 4 (“completely agree”).

The Mind Wandering Deliberate (MW-D) and the Mind Wandering Spontaneous (MW-S) are two 4-item self-reported questionnaires assessing the participants’ propensity to mind wander in daily life, i.e., trait MW (Carriere et al., 2013). Items are scored on a 7-point Likert scale ranging from 1 (“rarely”) to 7 (“often”).

The Daydreaming frequency scale (Giambra, 1993; Stawarczyk et al., 2012) is a 12-item self-reported questionnaire measuring the extent to which patients experienced MW and daydreams in daily life. Items are scored on a 5-point Likert scale, with greater scores indicating higher rates of MW.

The Self-Reported Wender-Reimherr Adult Attention Deficit Disorder Scale (SR-WRAADDS) (Marchant et al., 2015) is a 61-item self-reported questionnaire assessing the global ADHD symptomatology, including inattention, hyperactivity, disorganization and emotional dysregulation with the 30 first items, and its impact on patients’ daily life, including oppositional defiant behaviors (9 items, e.g., “being in conflict, looking for an argument”), additional symptoms (7 items; “having a confused mind,” “taking risks, being a daredevil,” “being confused with too many connections in the mind at the same time,” “having problems with the authorities,” “making unsuccessful efforts,” “having difficulties waking up” and “having minor physical symptoms such as headaches, stomach problems, muscle pain”), academic difficulties (4 items, e.g., “having difficulties to mobilize all skills or abilities at work”) and social skills (11 items, e.g., “having difficulties making and keeping friends”) with the 31 last items. A recent factor analysis led on the 30 symptomatic-related items in a sample of adults with ADHD yielded a four-factor model, encompassing: (i) attention/disorganization, (ii) hyperactivity/restlessness, (iii) impulsivity/emotional outbursts, (iv) emotional lability (Weibel et al., 2022).

### 2.3. Statistical analyses

Descriptive statistics included frequencies and percentages of categorical variables together with means and standard deviations of continuous variables. To test whether racing thoughts and MW are distinct phenomena in adult ADHD, a factor analysis with the principal component extraction method was performed. To simplify and clarify the data, a Varimax rotation with Kaiser normalization was applied. Factors were retained when their eigenvalue was greater than 1. Only non-overlapping items with loadings greater than 0.50 were considered as being part of a factor. Two multiple linear regression analyses were carried out. The first regression analysis was performed to partial out the specific contribution of ADHD symptoms on the extracted factors. The second regression analysis was led to determine the specific contribution of the extracted factor on ADHD functional impairment. These analyses were performed using the factor scores calculated by the software for each extracted component. Analyses were performed using the Jamovi® software (version 2.2.3.0).

## 3. Results

### 3.1. Demographic description

In our sample of 84 adults with ADHD, the combined presentation was the most prevalent, concerning 73.81% of the group, followed by the inattentive presentation and the hyperactive one (23.81 and 2.38% respectively). Regarding psychiatric comorbidities, 3.57% (*n* = 3) of patients had a current major depression episode, 32.14% (*n* = 27) had past major depression episode, 15.48% (*n* = 13) presented a comorbid, but stabilized bipolar disorder, 17.86% (*n* = 15) presented anxiety disorders and 7.14% (*n* = 6) were diagnosed with an autism spectrum disorder. 70.24% (*n* = 59) did not take any psychotropic drugs (Table 1).

### 3.2. Factor analysis

The factor analysis was performed on a set of 6 variables, comprising the three RCTQ-13 subscales (i.e., thought overactivation, burden of thought overactivation and thought overexcitability), the MW-S, the MW-D and the Daydreaming Frequency Scale. The analysis yielded two factors with eigenvalues greater than 1 (eigenvalues 2.84 and 1.22 respectively), explaining 47.47 and 20.29% of the variance. The first factor had strong factor loadings for the three RCTQ-13 subscales. By contrast, the second factor had strong factor loadings for the MW-D, the Daydreaming Frequency Scale and the MW-S (see Table 2). Given the results, we labeled factor 1 “*Racing/overactive thoughts*,” and factor 2 “*Mind wandering*.” Of note, we obtained the same two-factor model when patients with a comorbid current depressive episode and bipolar disorder were excluded from the analysis.

**Table 2:** Factor analysis.

	Factor 1	Factor 2
% Explained Variance	47.47%	20.29%
RCTQ-13 – Thought overactivation	0.838	0.126
RCTQ-13 – Burden of thought overactivation	0.895	0.093
RCTQ-13 – Thought overexcitability	0.884	0.052
MW-D	−0.088	0.713
Daydreaming frequency scale	0.196	0.791
MW-S	0.493	0.575

MW-D, mind wandering-deliberate; MW-S, mind wandering-spontaneous; RCTQ-13, racing and crowded thoughts questionnaire-13 items.

### 3.3. Multiple regression analyses

Multiple regression analyses were conducted to partial out the specific contribution of ADHD symptoms on both factors: “*Racing/overactive thoughts*” and “*Mind wandering*.”

The four symptomatic facets of ADHD identified in a recent factor analysis of the SR-WRAADDS (i.e., “attention/disorganization,” “hyperactivity/restlessness,” “impulsivity/emotional outburst” and “emotional lability”) were simultaneously entered as predictors in the model. Results are presented in Table 3. Regarding the “*Racing/overactive thoughts*” factor, the predictors accounted for 20.3% of the variance [*F*(4,74) = 5.96; *p* < 0.001] and “emotional lability” was the main predictor of scores (*β* = 0.25, *p* = 0.03). Regarding the “*Mind wandering*” factor, the regression model was not significant [*F*(4,74) = 0.35; *p* = 0.84]. Results of regression analyses were unchanged when comorbidities were entered as covariates in the analysis.

**Table 3:** Multiple regression analysis: contribution of ADHD symptoms to the obtained factors.

	Factor 1 – “Racing/overactive thoughts”^a^			Factor 2 – “Mind Wandering”^b^		
	*β*	*t*	*p*	*β*	*t*	*p*
SR-WRAADDS - Inattention/Disorganization	0.194	1.826	0.07	0.050	0.411	0.68
SR-WRAADDS - Hyperactivity/Restlessness	0.211	1.750	0.08	−0.071	−0.514	0.61
SR-WRAADDS - Impulsivity/Emotional outbursts	0.064	0.486	0.62	0.112	0.743	0.46
SR-WRAADDS - Emotional lability	0.253	2.202	0.03*	0.041	0.309	0.76

SR-WRAADDS, self-reported Wender Reimherr adult attention deficit disorder scale. *p < 0.05. ^a^*R*^2^ = 0.244, adjusted *R*^2^ = 0.203, *F*(4, 74) = 5.96, *p* < 0.001. ^b^*R*^2^ = 0.019, adjusted *R*^2^ = −0.034, *F*(4, 74) = 0.35, *p* = 0.84.

Multiple regression analyses were also conducted to partial out the specific contribution of thought phenomena on functional impairment. The “*Racing/overactive thoughts*” factor and the “*Mind wandering*” factor were entered as predictors in the models. Regarding the 4 subscales on ADHD functional impairment of the SR-WRAADDS, the regression models were significant for the “oppositional defiant behaviors,” the “additional symptoms” and the “academic difficulties” subscales (*p* < 0.05; see Table 4). Predictors accounted, respectively, for 5.96, 8.60 and 8.22% of the variance. In the three models, the “*Racing/overactive thoughts*” factor was the main predictor (*β* = 0.28, *p* = 0.01; *β* = 0.33, *p* = 0.004 and *β* = 0.32, *p* = 0.005).

**Table 4:** Multiple regression analysis: contribution of the obtained factor on daily functioning of adults with ADHD.

	SR-WRAADDS – oppositional defiant behaviors^a^			SR-WRAADDS – additional symptoms^b^			SR-WRAADDS – academic difficulties^c^			SR-WRAADDS – social skills^d^		
	*β*	*t*	*p*	*β*	*t*	*p*	*β*	*t*	*p*	*β*	*t*	*p*
“Racing/overactive thoughts” factor	0.279	2.54	0.317	0.326	3.01	0.004*	0.317	1.37	0.005*	0.177	1.59	0.12
“Mind Wandering” factor	0.085	0.78	−0.061	0.070	0.64	0.52	−0.061	−0.26	0.57	−0.155	−1.39	0.17

SR-WRAADDS, self-reported Wender Reimherr adult attention deficit disorder scale. *p < 0.05. ^a^*R*^2^ = 0.084 adjusted *R*^2^ = 0.060, *F*(2, 76) = 3.47, *p* = 0.036. ^b^*R*^2^ = 0.109 adjusted *R*^2^ = 0.086, *F*(2, 76) = 4.67, *p* = 0.012. ^c^*R*^2^ = 0.108 adjusted *R*^2^ = 0.082, *F*(2, 76) = 4.50, *p* = 0.01. ^d^*R*^2^ = 0.057 adjusted *R*^2^ = 0.032, *F*(2, 76) = 4.67, *p* = 0.107.

## 4. Discussion

This study is the first investigating both self-reported MW and racing thoughts in adults with ADHD. Using a factor analysis approach, we found that MW and racing thoughts are dissociable thought phenomena. Indeed, one factor encompassed the three RCTQ subscales (i.e., thought overactivation, burden of thought overactivation and thought overexcitability) whereas the other factor comprised the deliberate and spontaneous dimension of MW and the daily MW frequency measure. The two factors were therefore, respectively, labeled “*Racing/overactive thoughts*” and “*Mind wandering*.” Accordingly, both factors were found to be associated with distinct ADHD symptoms: emotional lability was the main predictor of the “*Racing/overactive thoughts*” factor (Weiner et al., 2018; Martz et al., 2021) whereas no ADHD symptoms contributed to the “*Mind wandering*” factor. In line with these results, only the “*Racing/overactive thoughts*” factor was found to be related to the reported functional difficulties of adults with ADHD.

First of all, the distinction between the “*Racing/overactive thoughts*” and “*Mind wandering*” factors can be explained by the conceptual underpinnings of both thought peculiarities. To date, MW episodes have been characterized according to: (i) their content, indicating whether the thoughts are related or not to the activity at hand (task-[un]related-thoughts) (Smallwood and Schooler, 2006; Smallwood and Schooler, 2015) and (ii) the intentionality of the MW episode (i.e., their deliberate or spontaneous occurrence) (Shaw and Giambra, 1993; Carriere et al., 2013; Seli et al., 2015, 2016, 2018). MW thus defines (un)intentional attention shifts resulting in “task-unrelated thoughts” (Shaw and Giambra, 1993; McVay and Kane, 2010; Seli et al., 2015; Smallwood and Schooler, 2015). This conceptualization of MW conveys the notion of control over the content of thoughts, which is part of the content-related dimension of self-generated thoughts (Christoff et al., 2016). By contrast, racing thoughts refer to an increased velocity and flow of thoughts, conveying the notion of a lack of control over thoughts’ generation and transitions, i.e., the dynamics of self-generated thoughts (Piguet et al., 2010; Weiner et al., 2018). Although this definition of racing thoughts differs from the content-related conceptualization of MW, more recent conceptualizations have also considered the dynamic transition of thoughts as part of MW (Christoff et al., 2016). The dynamics of self-generated thoughts relate to the constraints over the content and transitions of thoughts (Christoff et al., 2016). In adult ADHD, it has been hypothesized that diminished constraints exerted on thoughts result in excessive variability in thoughts’ movement (Christoff et al., 2016), akin to racing thoughts. Interestingly, in a recent experience sampling study, adults with ADHD reported that during MW episodes, their thoughts belonged to a looser structure flow of thoughts, in comparison to healthy subjects (Madiouni et al., 2020), suggesting that even when attention resources are allocated to internal concerns, thoughts might race and overlap. Consistent with our results, it can be hypothesized that MW and racing thoughts are not mutually exclusive, but are actually two co-occurring mental phenomena that may contribute to mental restlessness in adult ADHD.

The distinction between the “*Racing/overactive thoughts*” and “*Mind wandering*” factors is also supported by their respective links with the ADHD symptomatology. Our results showed that emotional lability contributed to the “*Racing/overactive thoughts*” factor. These findings are consistent with a prior study led by our team, in which elevated cyclothymic temperament traits, reflecting affective lability, and motor hyperactivity were found to be predictive of increased racing thoughts in adults with ADHD (Martz et al., 2021). Surprisingly, the ADHD symptomatology, including inattention, was not predictive of the “*Mind wandering*” factor. Yet, MW and especially spontaneous MW has been put forward as a hypothetical mechanism underlying attention impairments in adults with ADHD (Seli et al., 2015; Mowlem et al., 2016; Bozhilova Michelini et al., 2018). However, the “*Mind wandering*” factor in our study encompassed spontaneous and deliberate MW, and the latter has been found to be unrelated to ADHD (Franklin et al., 2014; Seli et al., 2015; Arabacı and Parris, 2018). This can be explained by the nature of the distractions that people with ADHD might experience. While MW corresponds to an internal distractibility decoupled from the perceptual world (Smallwood and Schooler, 2015), inattention symptoms in ADHD encompass more broadly external distractibility associated to disorganization (Becker et al., 2018; Biederman et al., 2019). Hence, it is possible that excessive MW corresponds to a cognitive comorbidity to the classic inattention symptoms in adult ADHD (Biederman et al., 2019), underpinned by different mechanisms. Indeed, MW and external distraction have been found to be distinct types of attention failures in studies with healthy subjects (Unsworth and McMillan, 2014; Varao-Sousa et al., 2018).

Similarly, regarding the functional impairment related to thinking abnormalities in ADHD, we found that racing/overactive thoughts predicted interpersonal problems, academic difficulties and additional ADHD symptoms (e.g., risk-taking and making unsuccessful efforts). By contrast, the “*Mind wandering*” factor was not linked to functional impairment. This result was surprising given that the literature sustains that beyond ADHD symptomatology, MW contributes significantly to the patients’ impaired quality of life (Biederman et al., 2019; Lanier et al., 2019). However, a difference has been established between deliberate and spontaneous MW, which correspond to two dissociable mental experiences (Seli et al., 2015, 2016). Indeed, deliberate MW episodes have been depicted as a beneficial experience, being initiated when it is appropriate and useful, such as during low-demanding tasks, to cope with boredom (Smallwood and Andrews-Hanna, 2013; Shepherd, 2019). Deliberate MW has also been found to be involved in idea incubation (Baird et al., 2012) or future planning (Smallwood and Andrews-Hanna, 2013; Shepherd, 2019). By contrast, spontaneous MW episodes occur mainly during demanding tasks (Smallwood and Andrews-Hanna, 2013), and may be considered as a failure of executive control, detrimental to cognitive performances (McVay and Kane, 2010; Seli et al., 2016; Shepherd, 2019). Importantly, in the factor analysis, the deliberate MW questionnaire had a greater factor loading than the spontaneous MW scale. Therefore, deliberate MW weighs more heavily in in the “*Mind wandering*” factor than spontaneous MW, explaining the absence of association with functional impairment. Instead, racing/overactive thoughts might be related to functional impairment because of the tight links between this kind of thinking abnormality and emotional lability (Weiner et al., 2018; Martz et al., 2021). Indeed, emotional dysregulation in adult ADHD is strongly associated with increased impairment in everyday life (Skirrow and Asherson, 2013; Surman et al., 2013; Shaw Stringaris et al., 2014), such as those measured here with the SR-WRAADDS (Weibel et al., 2022). As an example, regarding academic difficulties, it can be hypothesized that due to the failures adults with ADHD have had to face, this domain is associated with highly negative emotions. Moreover, it has previously been found that racing thoughts were not associated with decreased cognitive performances in ADHD (Martz et al., 2022). However, given the relationship between racing thoughts and emotion dysregulation, this symptom alone, and not MW, is related to self-reported functional impairment in adults with ADHD.

We acknowledge several limitations to this study. First, the questionnaires used to assess MW encompassed only its intentionality dimension. Future studies should aim at comparing racing/overactive thoughts to task-(un)relatedness and especially MW dynamics (Christoff et al., 2016; Mills et al., 2018). Second, this study was uniquely based on self-report measures. It would be relevant to compare MW and racing thoughts using more objective measures, such as cognitive tasks allowing to measure state-MW and experience sampling methods. Those further studies would be particularly relevant as MW and racing thoughts have been, respectively, associated with attentional failure and preserved cognitive flexibility (McVay and Kane, 2010; Mooneyham and Schooler, 2013; Martz et al., 2022). In addition, phenomenological qualitative analyses of patients’ reports of their thinking styles are needed to delineate the nuances of MW and racing thoughts in ADHD. These types of design might help to disentangle the two thought phenomena and determine whether they may co-occur and jointly contribute to mental restlessness in adults with ADHD. Third, this study focused on ADHD, and did not include a comparative sample of healthy subjects nor adults in a hypomanic episode of bipolar disorder. Indeed, although (hypo)manic patients are highly distractible (American Psychiatric Association, 2013; Brus et al., 2014), MW has been scarcely investigated in bipolar disorder.

In conclusion, this study highlighted for the first time that MW and racing thoughts might correspond to two dissociable thought peculiarities in adults with ADHD, both contributing to the feeling of mental restlessness. Importantly, given the associations between racing thoughts and the ADHD symptoms, but also the relationship between racing thoughts and the functional impairments, racing thoughts should be considered when investigating mental restlessness in adult ADHD.

## Data availability statement

The raw data supporting the conclusions of this article will be made available by the authors, without undue reservation.

## Ethics statement

This study, involving humans, was approved by Regional ethics committee of the South of France (CPP SUD MEDITERRANEE II; February 19, 2018; reference number: 2017-A01618-45). The study was conducted in accordance with the local legislation and institutional requirements. The participants provided their written informed consent to participate in this study.

## Author contributions

EM contributed to the study conception, performed the data collection and coding, the statistical analysis, and wrote the first draft of the manuscript. LW, AB, and SW contributed to the study conception, the statistical analysis and the manuscript revision. All authors approved the submitted version.

## Conflict of interest

The authors declare that the research was conducted in the absence of any commercial or financial relationships that could be construed as a potential conflict of interest.

## Publisher’s note

All claims expressed in this article are solely those of the authors and do not necessarily represent those of their affiliated organizations, or those of the publisher, the editors and the reviewers. Any product that may be evaluated in this article, or claim that may be made by its manufacturer, is not guaranteed or endorsed by the publisher.
